# *Aspergillus fumigatus* Gliotoxin Inhibits LC3‐Associated Phagocytosis in Macrophages in a Calcium‐Dependent Manner

**DOI:** 10.1155/jimr/5542735

**Published:** 2026-04-29

**Authors:** Yuqing Sun, Fangyan Chen, Ling Zhang, Caopei Zheng, Yu Wang, Yulin Zhang, Li Han

**Affiliations:** ^1^ Department of Respiratory and Critical Care Medicine, Beijing Youan Hospital, Capital Medical University, Beijing, China, ccmu.edu.cn; ^2^ Department for Disinfection and Infection Control, Chinese PLA Center for Disease Control and Prevention, Beijing, China, chinacdc.cn; ^3^ Laboratory for Clinical Medicine, Capital Medical University, Beijing, China, ccmu.edu.cn; ^4^ Beijing Research Center for Respiratory Infectious Diseases, Beijing, China

**Keywords:** *Aspergillus fumigatus*, calcium, gliotoxin, LC3-associated phagocytosis, MAPK10

## Abstract

As a pivotal secondary metabolite of *Aspergillus fumigatus*, gliotoxin (GT) has many toxicological effects on mammalian cells; however, its function on LC3‐associated phagocytosis (LAP) induced by *A. fumigatus* in macrophages is poorly understood. Here, it was found that pretreatment of macrophages with GT can significantly attenuate the conversion of LC3‐II. In parallel, the expression of Rubicon, the putative indicator of LAP in macrophages, was dampened in a similar trend. Loss of ability to produce GT made the conidia of *gliP*Δ mutant of *A. fumigatus* induce more LC3‐II conversion in THP1 cells, which could be inhibited by exogenous GT. Comparative transcriptomic analysis showed that GT can promote the expression of MAPK10, and the calcium‐release regulatory pathway was enriched between the differentially expressed genes. Further, GT promotes the release of ROS at a concentration of 50 ng/mL, and inhibition by GT on LC3‐II production in macrophages during *A. fumigatus* infection could be restored by pretreatment with calcium inhibitors but not affected by the inhibitors for JNK and siRNA for MAKP10. Collectively, our data demonstrated that GT exerts an inhibitory effect on LC3‐associated phagocytosis in macrophages via a calcium‐dependent mechanism, and MAPK10 exists downstream of LAP.

## 1. Introduction

*Aspergillus fumigatus* (*A. fumigatus*) is the pathogenic fungus that primarily invades immunocompromised hosts through opportunistic infections and can cause various manifestations of aspergillosis, with invasive pulmonary aspergillosis (IPA) being the most severe [1, 2]. *A. fumigatus* interacts dynamically with different host immune cells through cell surface agents or secreted agents. Some toxins, such as GT and other enzymes, are secreted. A study examined 12 biological metabolites of *A. fumigatus* strains, among which GT was the most toxic factor [3–5]. As a secondary fungal metabolite, gliotoxin (GT), a member of the thiopiperazine family, also possesses a disulfide bond spanning the piperazine ring and an aromatic amino acid, which is mainly produced by several opportunistic pathogenic *Aspergillus* species, with ~90% of GT production derived from *A. fumigatus* rather than from other *Aspergillus* species (the same for *Candida*) [6]. It has been reported that GT is majorly produced during the germination process of *Aspergillus* spores rather than by resting spores [7]. In addition, it was also reported that GT is present in the airways during *Aspergillus* colonization and exerts the biological significance of *Aspergillus* via various potential immunosuppressive mechanisms [8, 9]. For instance, GT plays a unique role in promoting cell apoptosis by triggering Bak, a member of the pro‐apoptotic Bcl‐2 family, exhibiting cytotoxicity to mammalian cells, leading to the production of reactive oxygen species (ROS), mitochondrial release of pro‐apoptotic factors, and activation of caspase‐3 [10–13]. It can also inhibit the phagocytosis of macrophages and the immune function of other cells. GT disrupts innate immune signaling by inhibiting proteasome‐mediated IκBα degradation, thereby blocking NF‐κB activation and subsequent pro‐inflammatory cytokine production [14]. Additionally, GT enhances fungal invasion by modulating host cell behavior: in pulmonary epithelial cells, it activates phospholipase D (PLD) and induces actin cytoskeleton rearrangement, facilitating the internalization of *A. fumigatus* conidia [15]. Beyond direct immunomodulation, GT suppresses angiogenesis, potentially impairing tissue repair and leukocyte recruitment during infection [16]. Critically, GT targets neutrophil function through dual pathways: it not only inhibits neutrophil migration, a key defense mechanism against fungal dissemination, but also disrupts nicotinamide adenine dinucleotide phosphate (NADPH) oxidase assembly, compromising ROS‐dependent microbial killing [17]. Previous studies have demonstrated that a concentration of 20 ng/mL of GT significantly inhibits the phagocytic activity of macrophages against *A. fumigates* [15]. Arias et al. [18] suggested that the inhibition of macrophage phagocytosis by GT may be mediated through interference with IP_3_ metabolism. Similarly, Schlam et al. [19] reported comparable findings, identifying that GT disrupts the regulation of downstream effectors of IP_3_, such as Rac and/or Cdc42. Moreover, GT‐induced inhibition of phagocytosis was found to be dependent on arachidonic acid but independent of cAMP [17].

As one of the major innate immune cells against *A. fumigatus* infection, macrophages play a crucial role in inhibiting fungal germination and promoting fungal killing [20, 21]. They perform phagocytic functions and eliminate pathogens as part of physiological processes [22]. After phagocytosis of *A. fumigatus* by macrophages, autophagy‐related mechanisms play an important role in targeting and degrading conidia by ultimately fusing them with lysosomes. Autophagy is an evolutionarily conserved process that enhances cellular and organismal survival in aquatic environments, significantly contributing to innate immunity [23]. Unc‐51‐like kin (ULK1) is the main component of the classical autophagy initiation complex, and its phosphorylation is a key mechanism for autophagy regulation [24]. The activity of the ULK1 complex is primarily inhibited by the mTOR signaling pathway. In contrast, the activation of the PI3K/Akt, NF‐κB, and AMPK pathways can enhance the autophagic capacity of macrophages, thereby facilitating the clearance of pathogens [25]. However, the main ULK1‐independent pathway is involved in clearing *A. fumigatus* infection in macrophages [26]. Intracellular macromolecular proteins and damaged organelles are mainly metabolized through bilayer membrane autophagosomes, promoted by autophagy [27]. Extracellular entities such as apoptotic cells and invasive pathogens are mainly processed through single‐layer membrane phagosomes, coordinated by LC3‐associated phagocytosis (LAP) [28]. LAP is a phagocytic mechanism that differs from classical autophagy in that it does not form a bilayer phagosome; instead, it creates a monolayer LAPosome. Additionally, LAP does not enhance the degradation capacity of the cell [29]. Previous studies have demonstrated that LAP can induce the phagocytosis of *A. fumigatus* and is essential for its clearance from the body [30]. Melanin produced by *A. fumigatus* enhances its pathogenicity by inhibiting LAP [28]. The LAP induced by *A. fumigatus* is regulated by multiple pathways; for instance, Tonia et al. showed that the LAP of *A. fumigatus* relies on the IL‐6 signaling pathway. The loss of IL‐6 signaling during sepsis results in impaired LAP activation, leading to immune dysregulation in monocytes and macrophages [31].

However, it is still unclear whether GT is involved in *A. fumigatus* conidia‐induced LAP in macrophages. The aim of this study was to explore the possible role of GT in *A. fumigatus* conidia‐induced autophagy during *A. fumigatus* infection.

## 2. Materials and Methods

### 2.1. Chemical Reagents, Antibodies, and siRNA

Chemical reagents: GT was purchased from DUOMI (Beijing, China). JNK inhibitor SP600125 and calcium inhibitors W7 and BAPTA‐AM were obtained from MedChemExpress (Shanghai, China). SiRNA for MAPK10 was from Sangon Biotech (Beijing, China). FITC‐phalloidin was purchased from Solarbio (Beijing, China).

Antibodies: anti‐GAPDH (5174, rabbit polyclonal, 1:1000) and anti‐ULK1 (8054, rabbit monoclonal, 1:1000) were purchased from Cell Signaling Technology (Maryland, USA); anti‐LC3 (14600‐1‐AP, rabbit polyclonal, 1:1000), anti‐P62/SQSTM1 (18420‐1‐AP, rabbit polyclonal, 1:1000), anti‐Rubicon (21444‐1‐AP, rabbit polyclonal, 1:1000), anti‐VPS34 (12452‐1‐AP, rabbit polyclonal, 1:1000), and anti‐MAPK10 (17572‐1‐AP, rabbit polyclonal, 1: 1000) were obtained from Proteintech (Wuhan, China); HRP‐conjugated goat anti‐rabbit IgG antibody (7074, 1:4000) was purchased from ZSGB‐BIO (Beijing, China).

### 2.2. *A. fumigatus* Strains and Cell Lines

*A. fumigatus* strains: *A. fumigatus* wild‐type strain B5233, its mutant strain *gliP*Δ (deletion of the peptide synthetase *gliP* gene), and the *gliP* reconstituted strain *gliPR* were gifts from Dr. KJ. Kwon Chung (National Institute of Health, Bethesda, Maryland) [32]. GT levels of *A. fumigatus strains’* supernatants were detected via targeted metabolomics (Figure S1). All of the *A. fumigatus* strains were propagated on Sabouraud dextrose agar (10 g/L peptone, 10 g/L glucose, 15 g/L agar, pH 6.0) for 5–8 days at 37 °C and prepared as described in a previous study [33].

Cell lines: The THP‐1 cell line (human acute monocytic leukemia cells) and the mh‐s cell line (mouse alveolar macrophages) were obtained from ATCC (America Type Culture Collection, TIB‐202, CRL‐2019) and cultured in RPMI‐1640 (GIBCO, Germany) supplemented with 10% heat‐inactivated fetal calf serum, 100 U/mL streptomycin, and 100 U/mL penicillin at 37°C in an atmosphere of 5% CO_2._ The THP‐1 cells were induced into a macrophage‐like phenotype through exposure to 100 ng/mL phorbol myristate acetate (PMA, Sigma–Aldrich, Germany) for 48 h for further experimentation. J774 cell line (mouse mononuclear macrophages) were obtained from ATCC (American Type Culture Collection, TIB‐67) and cultured in DMEM (GIBCO, Germany) supplemented with 10% heat‐inactivated fetal calf serum, 100 U/mL streptomycin, and 100 U/mL penicillin at 37°C in an atmosphere of 5% CO_2_.

### 2.3. Targeted Liquid Chromatography–Tandem Mass Spectrometry (LC–MS/MS) Analysis of Gliotoxin

GT in cell culture supernatants was quantified using a validated targeted LC–MS/MS method. Briefly, supernatants were collected, centrifuged to remove debris, and stored at −80°C. For analysis, 100 µL of supernatant was mixed with 200 µL methanol, vortexed, and centrifuged (12,000 rpm, 10 min); the clear supernatant was injected into the LC–MS/MS system.

Chromatographic separation was performed on a C18 column (100 × 2.1 mm^2^, 2.5 µm) with a mobile phase of 0.1% formic acid in water (A) and methanol (B) at 0.35 mL/min using a 5 min gradient. Detection was carried out on a triple‐quadrupole mass spectrometer with electrospray ionization in positive mode and multiple reaction monitoring. GT was monitored using the transitions m/z 327.1–245.0 and 327.1–263.0.

Calibration standards (2.5–1000 ng/mL) and quality‐control samples were prepared in matrix and processed together with study samples. The method showed good linearity (*r* > 0.99) and acceptable accuracy and precision (within ±15%) in accordance with current bioanalytical guidelines.

### 2.4. Preparation of the Conidia

*A. fumigatus* conidia were harvested and prepared in the same way as previously studied [33]. After 5–8 days of cultivation, the conidia of *A. fumigatus* were scraped from the agar plate using phosphate‐buffered saline supplemented with 0.1% Tween 20 (PBST). Then resuspend it in sterile 0.1% PBST. Pass the conidia through a 40 µm cell filter to remove hyphal fragments. Subsequently, they were counted on a hemacytometer, and the resting conidia were washed twice with sterile 0.1% PBST and stored at 4°C for use within 48 h. To prepare swollen conidia, resting conidia were incubated in a liquid Sabouraud medium at 37°C for a specified period of time. The preparations that were incubated for 6 h contained swollen conidia and early germlings with <5 µm hyphal extensions.

### 2.5. Cell Cytotoxicity Assay

The cells were seeded in 96‐well plates (1 × 10^5^cells/well)and treated with different concentrations (0, 12.5, 25, 50, 100, 200 ng/mL) of GT for 2 h, or treated with 25 ng/mL GT for different time (0, 0.5, 1, 2, 4, 6, 8 h) according to grouping. After that, the cell viability was determined by Cell Counting Kit‐8 (CCK‐8) according to the manufacturer (DOJINDO company, Beijing, China). Specifically, add 10 μL of CCK‐8 solution to each well; incubate the culture plate in an incubator (37°C, 5% CO_2_) for 2 h. In addition, the absorbance at 450 nm was measured with the microplate reader. For each independent experiment, six technical replicate wells were included per condition to account for pipetting and measurement variability. Statistical analyses were performed using the mean value of the six technical wells as one biological replicate, and a total of three independent biological replicates (*n* = 3) were performed on different days.

### 2.6. Western Blotting

Cells were seeded in a 6‐well plate and cultured overnight. After exposure to GT or *A. fumigatus* conidia with or without the indicated pharmacological agent pretreatment, the cells were washed with cold PBS. For silencing expression of MAPK10, cells were preincubated with 20 µM JNK inhibitor SP600125 for 1 h before stimulation. For blocking the release of calcium, cells were preincubated with calcium inhibitor 10 µM W7 for 1 h or 10 μM BAPTA‐AM for 1 h before stimulation. Then, the cells were lysed in cold RIPA lysis buffer containing phosphatase inhibitors and protease inhibitors (Beyotime, Shanghai, China). After centrifugation (13,000 rpm, 20 min), the supernatant was collected as the total cellular protein extract. The total protein concentration was determined by BCA assay kit (Biomed, Beijing, China). Equal amounts of protein (40 μg) were separated by SDS–PAGE electrophoresis and transferred to a polyvinylidene difluoride (PVDF) membrane (Merck Millipore, Germany). The membrane containing the targeted protein was incubated with the indicated primary antibodies at room temperature for 2 h or 4°C overnight and subsequently with the HRP‐conjugated secondary antibody at room temperature for 1 h. The proteins were visualized using ECL reagent (Pierce ECL Western Blotting Substrate, Thermo Scientific). Specifically, we used ImageJ software to analyze the gray values of the bands, following the analysis procedure as follows: Open the file_Subtract Background_Outline the target protein_ Analyze _Gels_Select First Lane_Gels_Plots Lanes_Identify the peaks generated in the software_Click on each peak one by one using the Magic Wand Tool_Obtain the gray values. Calculate the ratio of the gray value of the target protein to that of GAPDH in the corresponding group, and finally normalize the ratios using the control group as the reference. Three independent biological replicates were conducted (The *p*‐value was calculated with *n* = 3).

### 2.7. Quantitative Reverse Transcription Polymerase Chain Reaction (qRT‐PCR)

Total RNA was extracted from cells using TRIzol reagent (Invitrogen), and the first‐strand complementary DNA (cDNA) was synthesized using the RevertAid First Strand cDNA Synthesis Kit (Tian Gen, Beijing, China). Briefly, cDNA was synthesized from 1 μg of isolated total RNA, oligo‐dT18, and reverse transcriptase in a final volume of 20 μL. Then, the cDNA was quantified with a SYBR Green Real‐Time PCR Master Mix Kit (Tian Gen, Beijing, China). A volume of 1 µL of specific forward and reverse primers (10 µM), Taqase, and 2 µL cDNA synthesis were mixed and performed with 40 thermocycles for 30 s at 94°C, 5 s at 94°C, and 30 s at 60°C. PCR quantification was conducted using the 2^−ΔΔCT^ method and normalized to GAPDH. For each experimental condition, three independent biological replicates were performed, and each PCR reaction was run in technical triplicate to ensure reproducibility.

### 2.8. RNA‐Seq Analysis

The number of cells in each sample was at least 1 × 10^6^, with three replicates in each group. RNA‐seq was completed in the same way as previously studied [34]. Briefly, the total RNA of cells was extracted with RNeasy Mini Kit (cat.74134; Qiagen). The integrity of RNA was detected by ultramicro ultraviolet–visible spectrophotometer, and the quality of RNA was measured with Qubit Fluorometer (Invitrogen, Carlsbad, CA). The cDNA libraries were constructed using VAHTS Universal V6 RNA‐seq Library Prep Kit for Illumina (Catalog No. NR604‐01, Vazyme Biotech Co. Ltd), and the experiment process is briefly introduced as follows: First, we used the VAHTS mRNA Capture Beads (Vazyme #N401) to enrich poly(A) + mRNA from the total RNA, and then removed rRNA used Ribo‐off rRNA Depletion Kit (Vazyme #N406), and randomly fragmented purified mRNA into ~250 bp in length. The mRNA was reverse transcribed into double‐stranded cDNA, adapters and tags were added, and the cDNA library was amplified. Finally, the samples were subjected to Illumina sequencing.

Primary sequencing data produced by Illumina HiSeq 2000 is called raw reads. Before doing any further analysis, quality control was required to detect whether the data were qualified. In addition, filtering of raw data was needed to decrease data noise. Several software updates were used to perform these tasks. After filtering, the remaining reads were called “clean reads” and used for downstream bioinformatics analysis. Clean reads were mapped to a reference sequence using *SOAPaligner/SOAP2*. No more than five mismatches were allowed in the alignment. The gene expression level was calculated by using the RPKM method (Reads per kilobase transcriptome per million mapped reads), and the *p*‐value was calculated according to the Poisson distribution. We used *p*‐value < 0.05 and the fold change ≥1.5 as the threshold to judge the significance of gene expression difference. Further, we used the DAVID online tool (http://david.abcc.ncifcrf.gov) to perform Gene Ontology (GO) enrichment analysis and KEGG Pathway enrichment analysis.

### 2.9. Confocal Microscopy

THP‐1 cells were seeded onto coverslips in 24‐well plates (5 × 10^4^ cells/mL) and grown for 24 h, and then were induced into a macrophage‐like phenotype through exposure to 100 ng/mL PMA for 48 h. After treatment with the indicated GT or *A. fumigatus* conidia, the cells were fixed in ice‐cold 4% paraformaldehyde/PBS at room temperature for 20 min. Then, the cells were permeabilized with 0.05% Triton X‐100/PBS at 4°C for 15 min. The samples were blocked in 5% bovine serum albumin (BSA) at room temperature for 30 min and incubated with primary antibody (anti‐LC3, 14600‐1‐AP, rabbit polyclonal, 1:500) overnight and then with fluorochrome‐conjugated secondary antibody (TRITC‐coupled goat anti‐rabbit IgG, 1:1000, red) for 1 h. The conidia of *A. fumigatus* were stained with a molecular probe (code F432, 1:100 dilution, Invitrogen) bound to fluorescein isothiocyanate (FITC, green) for 1 h. Additionally, 5 µM FITC‐phalloidin was added for 30 min. Then, DAPI (4′,6‐diamidino‐2‐phenylindole) was added for 5 min to stain the nucleus (blue). The preparations were observed under a laser scanning confocal microscope (Olympus FluoView FV 1000). The images were processed using Nikon‐Eclipse‐Ti (Japan). For each biological replicate, 3–5 randomly selected fields were imaged per condition, avoiding overlapping areas and ensuring unbiased sampling. All raw images were processed using ImageJ without nonlinear adjustments. Mean fluorescence intensity of the entire field (not individual cells) was measured using ImageJ. A total of three independent biological replicates (*n* = 3) were performed for each imaging assay.

### 2.10. Measurement of Intracellular Calcium

For microplate‐based calcium measurements, cells were seeded in 96‐well clear‐bottom black plates (1 × 10^5^ cells/well) and cultured overnight. The cells were stimulated with GT (25 ng/mL) for 2 h and incubated with 5 mM Fluo‐4 AM (J&K, China) diluted in PBS for 30 min. After the staining of Fluo‐4 AM, the cells were washed with PBS three times. Then, the intracellular calcium concentrations were determined by detecting the fluorescence value using a microplate reader(excitation wavelength = 494 nm, emission wavelength = 516 nm). The mean fluorescence value of the six wells was used as the biological replicate value for statistical analysis. A total of three independent biological replicates (*n* = 3) were performed on different days.

For flow cytometry analysis, cells were seeded in 6‐well plates (1 × 10^6^ cells/well) and cultured overnight. After stimulation with GT (25 ng/mL) for 2 h, the culture supernatant was removed, and the cells were treated with trypsin for 1 min; the cell suspension was collected into a 15 mL sterile centrifuge tube. After centrifugation (800 rpm, 5 min), the cell pellets were resuspended in 0.5 mL PBS and incubated with 4 μM Fluo‐4 AM for 1 h at 37°C. The cells were washed three times with PBS and resuspended in 1 mL PBS. Total cells were examined with a BD FACSCanto II flow cytometer and analyzed by FlowJo V10 software. For each independent experiment, three wells were processed in parallel; however, because all cells are pooled into one suspension before acquisition, the flow cytometry measurement represents one biological replicate per experiment.

### 2.11. ROS Measurement

THP1 cells were seeded at a density of 1 × 105/well in a 96‐well plate. After induction of differentiation, THP1 cells were treated with 0, 25, 50, 100, and 200 ng/mL GT for 2 h and 25 ng/mL mycotoxin for 0, 2, 4, 6, and 8 h. As for the group of calcium pretreatment, we used 25 ng/mL GT to stimulate 2 h after the calcium inhibitor. Then, 10 μM staining solution was added to 200 μL and incubated at 37°C for 30 min. The staining solution was removed and washed with PBS twice. Fluorescence intensity was detected using a microplate reader at an excitation wavelength of 518 nm and an emission wavelength of 594 nm. For each biological replicate, five technical wells were included per condition. The mean fluorescence intensity of the five wells was used as the biological replicate value. A total of three independent biological replicates (*n* = 3) were performed on separate experimental days.

### 2.12. GSH Measurement

THP1 cells were seeded at a density of 1 × 106/well in a 6‐well plate. After induction of differentiation, they were intervened with 25 or 50 ng/mL GT for 2 h. The cells were collected and resuspended in 1 mL of detection reagent 1 (Solarbio, BC1175). After rapid freezing in liquid nitrogen, they were melted at 37°C three times and centrifuged at 12,000 g for 10 min at 4°C. The supernatant was removed and placed on ice for testing. Add 20 μL of the test sample, 140 μL of detection reagent 2 (Solarbio, BC1175), and 40 μL of detection reagent 3 (Solarbio, BC1175) according to the instructions. Mix well and let it stand for 2 min. Then, use a microplate reader to detect the OD value at 412 nm. For each biological replicate, five technical wells were included per condition. The mean fluorescence intensity of the five wells was used as the biological replicate value. A total of three independent biological replicates (*n* = 3) were performed on separate experimental days.

### 2.13. Statistical Analysis

All experiments were repeated at least three times independently. Differences between the two groups were evaluated by Student’s *t*‐test, and one‐way analysis of variance (ANOVA) was used to analyze multiple comparisons among multiple exposure groups. Data are presented as mean ± SEM of biological replicates. All statistical analyses were performed with GraphPad Prism 9.5.1. For enhanced transparency, exact *p* values are provided directly in the figures.

## 3. Results

### 3.1. GT Attenuates the Conversion of LC3‐II in Macrophages in a Time‐ and Dose‐Dependent Manner

Insufficient activation of LAP may affect the control of *A. fumigatus* infection; however, the effect of GT on the autophagy in host cells has rarely been explored, especially in macrophages, which serve as the first line of immune defense in the body. Thus, we first investigated the impact of GT on the LAP. GT is generally believed to have a promoting effect on cell apoptosis and may have an impact on cell viability. Our previous study showed that pretreatment with 50 ng/mL GT for 4 h markedly affected cell morphology but not the viability of A549 alveolar epithelial cells or J774 macrophages. To clarify the effect of GT on the cell viability of THP‐1 cells in vitro, the CCK‐8 assays were performed. It was found that treatment of 50 ng/mL or less of GT did not significantly affect the cell viability of THP‐1 cells in less than 2 h (Figure S2A, B). We then stained the F‐actin cytoskeleton of THP1 cells with FITC‐labeled phalloidin to visualize cell morphology. The results showed that GT stimulation weakened the fluorescence intensity and disrupted the integrity of F‐actin (Figure S2C, D).

**Figure 1:**
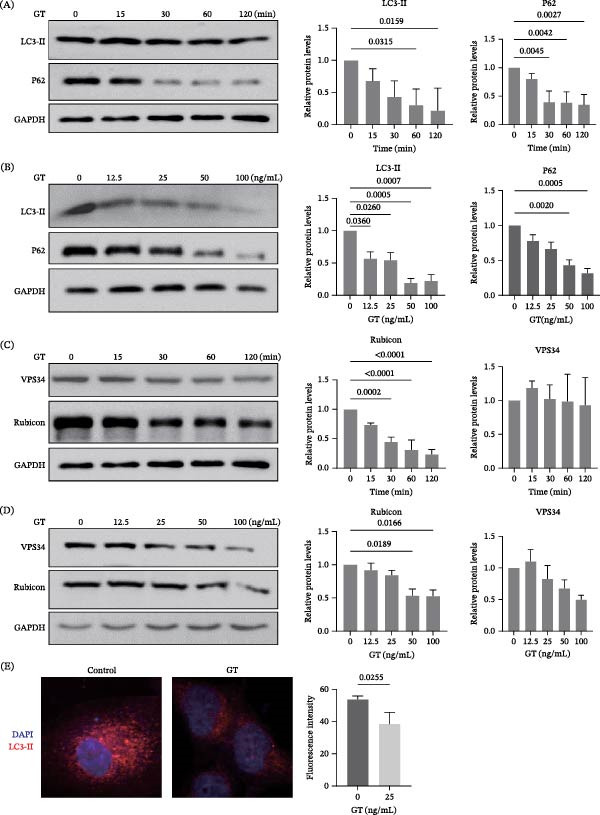
GT attenuates LAP and in THP1 cells. (A and B) Western blot analysis of LC3‐II and SQSTM 1/p62 in THP1‐derived macrophages treated with gliotoxin (GT, 25 ng/mL) for the indicated time points (0–120 min). LC3‐II and SQSTM 1/p62 accumulation were reduced in a time‐dependent manner. (C and D) THP1‐derived macrophages were exposed to increasing concentrations of GT (0–100 ng/mL) for 2 h. LC3‐II and SQSTM 1/p62 levels decreased in a dose‐dependent fashion. Densitometric quantification of LC3‐II was normalized to GAPDH. (E) Representative confocal micrographs showing LC3 fluorescence (red) in THP1 cells treated with 25 ng/mL GT for 2 h. Nuclei were counterstained with DAPI (blue). GT markedly reduced LC3 puncta formation compared with untreated controls. All experiments were independently repeated at least three times. GAPDH loading controls in (A) and (C) were derived from the same membrane.

The conversion of LC3‐II and expression of three key modulators on the LAP signaling pathway, p62, Rubicon, and VPS34 in THP‐1 cells were detected by western blot after the treatment with 25 ng/mL GT for different time periods or different concentrations of GT for 2 h. As shown in Figure 1A, B, the level of LC3‐Ⅱin THP‐1 cells decreased in a time‐ and dose‐dependent manner after exposure to GT, and so did the expression of both Rubicon and p62 in THP‐1 cells (Figure 1C, D). However, the expression of VPS34 in THP‐1 cells did not change significantly with the treatment of 25 ng/mL GT for 2 h (Figure 1C). Even under the higher concentration, 50 ng/mL or 100 ng/mL of GT, the expression of VPS34 had only a slight decrease without any significant difference (Figure 1D). Moreover, under the laser confocal microscopy, the level of LC3‐II marked by red fluorescence was significantly lower in THP‐1 cells stimulated by 25 ng/mL GT compared to that in the unstimulated cells (Figure 1E and Figure S3). These data indicated that GT could inhibit the LC‐3II conversion in a time‐ and dose‐dependent manner in THP‐1 cells. To further confirm the inhibitory effect of GT on LAP in macrophages, we stimulated mouse mononuclear macrophages J774 and mouse alveolar macrophages mh‐s with different concentrations of GT and found that GT inhibited LC3‐II transformation and Rubicon expression of J774 and mh‐s in a concentration‐dependent manner (Figure S4).

### 3.2. GT Suppresses LC3‐Associated Phagocytosis Induced by *A. fumigatus* in Macrophages

**Figure 2:**
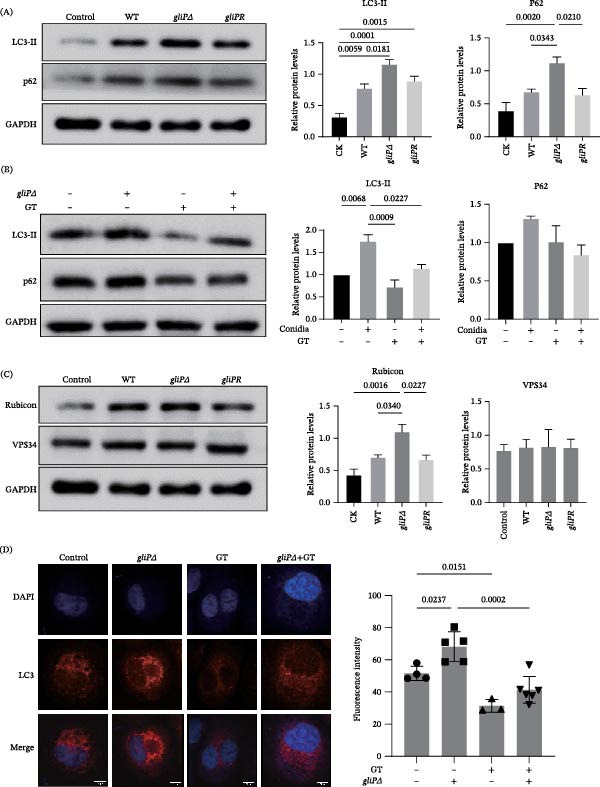
GT attenuates *A. fumigatus*‐induced LAP in THP1 cells. (A and C) THP1‐derived macrophages were infected with WT, *gliP*Δ, or *gliP*R swollen conidia of *A. fumigatus* (MOI = 5) for 2 h. Western blot and quantification of LC3‐I/II showed that LAP activation was significantly attenuated in cells infected with WT or *gliP*R *A. fumigatus* compared to *gliP*Δ strains. (B and D) THP1‐derived macrophages were infected with WT and *gliP*Δ conidia (MOI = 5) in the presence or absence of GT (25 ng/mL) for 2 h. GT was added simultaneously with conidia. Western blot and confocal microscopy revealed that GT suppressed the LC3‐II conversion and SQSTM1/p62 expression induced by *gliP*Δ infection. All experiments were performed independently at least three times. GAPDH loading controls in (A) and (C) were derived from the same membrane.

Previous studies have shown that *A. fumigatus* conidia can induce the recruitment of LC3‐Ⅱ in macrophages [30]. It was interesting to know whether GT regulates or affects this process. To test the role of GT, *A. fumigatus* wild‐type strain B5233 (WT), *gliP*Δ (*gliP* gene‐knockout mutant), which could not synthesize GT, and *gliPR* mutant (*gliP* gene complemented) were used to stimulate the THP‐1 cells. As illustrated in Figure 2A, the level of LC3‐II in THP‐1 cells was upregulated by the stimulation of the WT strain of *A. fumigatus*, and so was the expression of p62 in THP‐1 cells. Intriguingly, the level of LC3‐II could be further promoted by the stimulation of the *gliP*Δ mutant but not by *gliPR*. A similar trend of change was also found in the expression of p62 (Figure 2A). Noteworthy, exogenous addition of GT significantly inhibited the increase of LC3‐Ⅱ induced by the *gliP*Δ mutant, and the alteration of p62 expression was consistent with that of LC3‐Ⅱ, although there was no significant difference between the groups (Figure 2B). Furthermore, the *gliP*Δ mutant markedly enhanced Rubicon expression induced by WT strains and the *gliP*R mutant, which was as similar as the trend of LC3‐Ⅱ; however, the expression of VPS34 had no obvious difference between the groups (Figure 2C). Lastly, under the laser confocal microscopy, it was illustrated clearly that the *gliP*Δ mutant induced a stronger red‐labeled LC3‐II signal compared to the control group; in contrast, GT blocked not only the basal LC3‐II conversion, but also the increase of LC3‐II conversion induced by *gliP*Δ mutant in THP‐1 cells (Figure 2D). These findings indicated that GT might play a negative role in the LAP process during *A. fumigatus* infection into macrophages.

### 3.3. Inhibition of LAP by GT is Independent of MAPK10 in Macrophages

**Figure 3:**
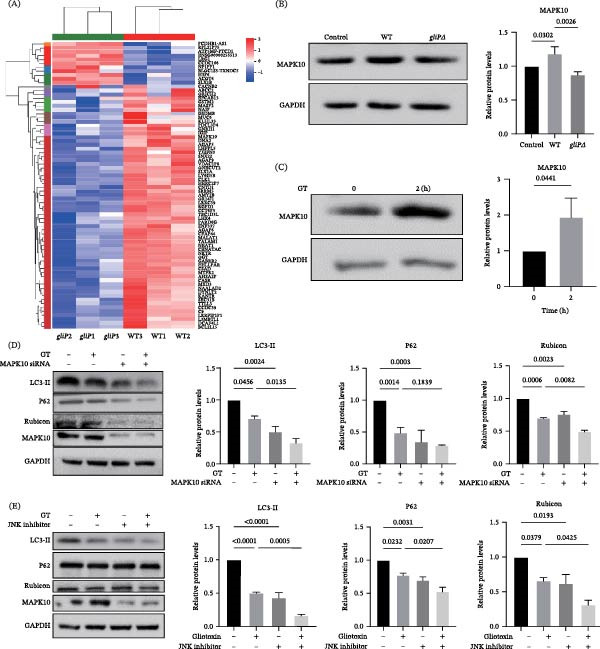
GT inhibits LAP in THP1 cells via a MAPK10‐independent manner. (A) Heatmap of differentially expressed genes (DEGs) in THP1 cells treated with *gliP*Δ compared to WT (MOI = 5, 2 h). The color scale represents log_2_ fold changes in gene expression relative to control cells. (B) MAPK10 protein levels in THP1 cells infected with wWT, *gliP*Δ or *gliP*R swollen conidia of *A. fumigatus* (MOI = 5) for 2 h. (C) Western blot analysis of MAPK10 expression in THP1 macrophages after GT (25 ng/mL, 2 h) treatment. (D and E) THP1 cells were transfected with MAPK10 siRNA for 48 h or pretreated with JNK inhibitor (SP600125, 20 μM, 1 h), followed by GT (25 ng/mL) exposure for 2 h. Western blot analysis and densitometric quantification of LC3, SQSTM1/p62, and Rubicon showed that GT inhibited LAP regardless of MAPK10 or JNK inhibition. All experiments were performed independently at least three times.

To further investigate the possible mechanism of inhibition of LAP by GT, comparative transcriptomic analysis was first performed in THP‐1 cells stimulated by either WT *A. fumigatus* conidia or *gliP*Δ mutant for 2 h, and the cluster analysis of differentially expressed genes (DEGs) was shown in the heatmap. Because it was previously reported that MAPKs might be involved in the regulation of autophagy, the MAPKs were first checked in the DEGs analysis (Figure 3A, Figure S5), and it was found that the expression of a MAPK, MAPK10 (also called JNK3) was down‐regulated by 1.5 times in *gliP*Δ‐stimulated cells compared to the cells stimulated by the wild‐type strain (Figure 3A). Further, by qRT‐PCR and western blot analysis, it was confirmed that the mRNA level and expression of MAPK10 in THP1 cells infected by the *gliP*Δ group were significantly lower than those of the wild‐type strain (Figure 3B and Figure S6A). Moreover, treatment of THP‐1 cells by exogenous 25 ng/mL GT for 2 h significantly improved the expression of MAPK10 in the cells (Figure 3C and: Figure S6B). These results hinted that GT might induce the expression of MAPK10 in macrophages.

Subsequently, it was speculated whether GT suppressed LAP by MAPK10 signaling. As shown in Figure 3D, the obvious repression of MAPK by transfection of its specific siRNA significantly suppressed the conversion of LC3‐II and the expression of p62 and Rubicon (Figure 3D). Interestingly, this repression of MAPK10 by siRNA could deepen the inhibition of GT on LC3‐II conversion and expression of p62 and Rubicon (Figure 3D). Moreover, because no specific inhibitor for MAPK10 was available, a universal inhibitor, SP600125, for the JNK family, which includes MAPK10, was used to investigate further the role of MAPK10 in GT‐inhibited LAP. As shown in Figure 3E, consistent with the effects of siRNA for MAPK10, pretreatment by SP600125 significantly inhibited not only the basal but also the GT‐suppressed conversion of LC3‐II in THP‐1cells (Figure 3E). Comparatively, no significant effect was found on the expression of p62 and Rubicon. These results suggested that the inhibition of LAP by GT might not be associated with MAPK10 because GT might promote MAPK and, subsequently, the LC3‐II conversion through some unknown mechanism.

### 3.4. GT Induces Oxidative Stress at a Certain Concentration in Macrophages

Moreover, the principal component analysis on the comparative transcriptomic data showed significant differences between the groups stimulated by the wild‐type strain and the *gliP*Δ mutant (explaining 34.91% of the variation) (Figure 4A).

Figure 4GT induces oxidative stress at a certain concentration in macrophages. (A) Principal component analysis (PCA) of transcriptomic data from THP1 cells treated with gliotoxin (GT, 25 ng/mL, 2 h). PC1 accounted for 34.91% of total variance, indicating a distinct transcriptional response to GT exposure. (B) Gene Ontology (GO) enrichment analysis of differentially expressed genes showing significant enrichment in oxidative stress–related biological processes. (C and D) THP1 macrophages were treated with GT (25 or 50 ng/mL) for 2 h. mRNA expression of *NOX2*, *p22^phox*, and *p40^phox* was measured by qRT‐PCR. Intracellular GSH levels were determined using a commercial detection kit by measuring absorbance at 412 nm. (E) Intracellular ROS levels were quantified using a fluorescence microplate reader (excitation 518 nm, emission 594 nm) after exposure to increasing GT concentrations (0–200 ng/mL). All experiments were performed independently at least three times.

**Figure:**
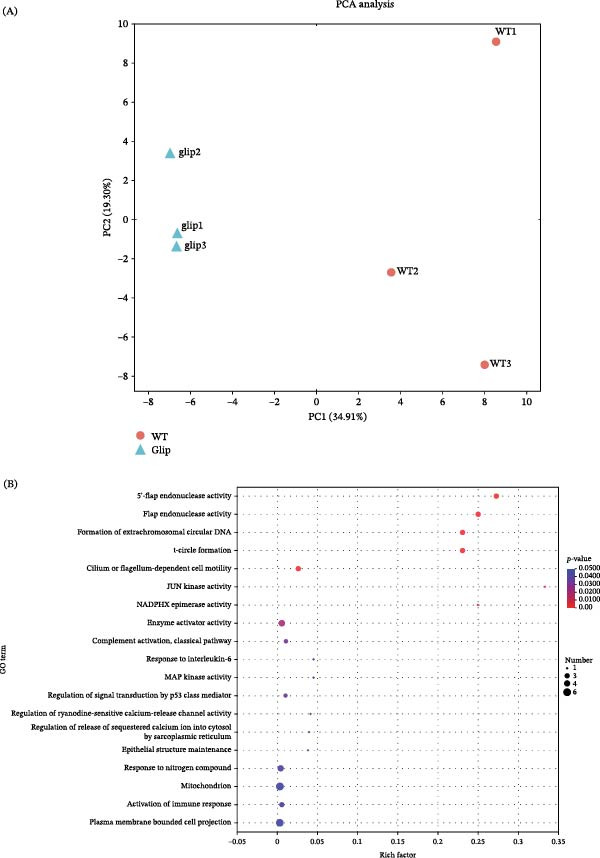

**Figure:**
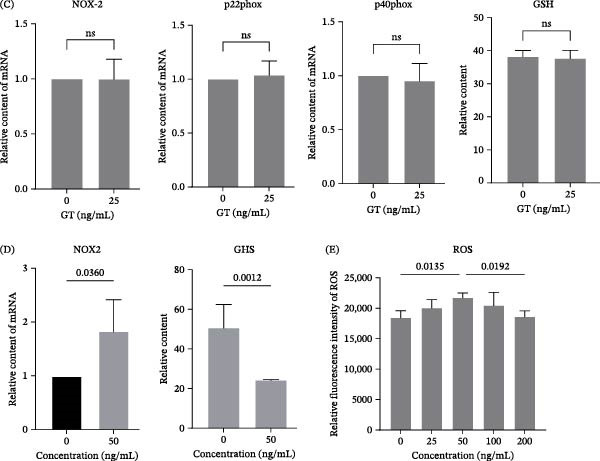

It is known that the LAP process requires NADPH oxidase 2 (NOX2) [26]. By GO enrichment analysis on the comparative transcriptomic data, it was found that genes of NADPHX epimerase activity were lower in *gliP*Δ‐stimulated THP1 cells compared to the cells stimulated by the wild‐type strain (Figure 4B). Further, it was shown that the expression of NOX2, p22phox, and p40phox could be improved, and the content of glutathione (GSH) was reduced by 50 ng/mL GT. In contrast, they were not affected by 25 ng/mL GT (Figure 4C, D). Furthermore, the intracellular ROS content could be significantly promoted by GT in the concentration of 50 ng/mL but not 25 ng/mL in macrophages (Figure 4E, Figure S7). Although Figure 4E includes higher GT concentrations to illustrate the dose–response pattern, these cytotoxic doses do not affect the interpretation of our ROS results at viability‐preserving levels. These results indicated that the ROS signal seemed not to be involved in the regulation of LAP by 25 ng/mL GT.

### 3.5. Involvement of Calcium Signal in GT‐Inhibited LAP Process in Macrophages

**Figure 5:**
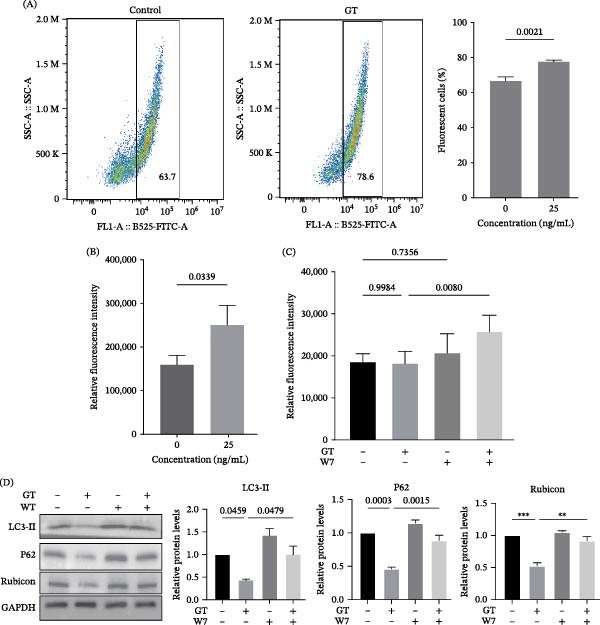
Calcium involved in the decreased LAP of THP1 cells exposed to GT. (A and B) THP1 macrophages were treated with GT (25 ng/mL) for 2 h. Intracellular calcium concentrations were determined using a fluorescence microplate reader and flow cytometry (excitation 494 nm, emission 516 nm). (C) THP1 cells were treated with GT (25 ng/mL, 2 h), calcium chelator W7 (10 μM, 1 h), or both. Intracellular ROS levels were measured fluorometrically to assess the contribution of calcium signaling to GT‐induced oxidative stress. (D) THP1 macrophages were treated with GT (25 ng/mL, 2 h), W7 (10 μM, 1 h), or both. Western blot analysis and densitometric quantification of LC3‐II, SQSTM1/p62, and Rubicon expression demonstrated that calcium inhibition partially reversed GT‐induced suppression of LAP. All experiments were independently repeated at least three times.

Because it had been reported that inhibition of calcium channels leads to the promotion of LAP in macrophages [35], and the results of RNA‐seq showed that there were two differentially expressed genes related to calcium signaling, *CACNB2* (calcium voltage‐gated channel auxiliary subunit beta 2) and *CASR* (calcium‐sensing receptor) (Figure 3A) in *gliP*Δ‐stimulated THP1 cells compared to the cells stimulated by the wild‐type strain, it was interesting that we investigated the possible role of calcium signaling in GT‐mediated LAP inhibition. First, it was found that the intensity of fluorescence labeling intracellular calcium with flou‐4 AM in THP1 cells was enhanced after the treatment with 25 ng/mL GT (Figure 5A), and the proportion of cells containing the fluorescence‐labeled calcium also significantly increased in the flow cytometry assay (Figure 5B). These results suggested that GT might induce the cytoplasmic release of calcium in THP1 cells.

Further, W7, as a selective calmodulin antagonist, was utilized to investigate the possible role of calcium signals in GT‐inhibited LAP processes. As shown in Figure 5C, pretreatment of THP1 cells with W7 could induce the release of intracellular ROS, and this effect was more pronounced in the 25 ng/mL GT‐stimulated group (Figure 5A). Pretreatment of THP1 cells with W7 alone could largely promote the conversion of LC3‐II; most importantly, it could restore the GT‐inhibited level of LC3‐II by GT. Similarly, inhibition by GT on the expression of either p62 or Rubicon was also recovered totally by pretreatment with W7 (Figure 5D, Figure S8). These results demonstrated that calcium might be a key negative regulator on the inhibition of LAP by GT in macrophages. To further determine the role of calcium, we pretreated THP1 cells with the intracellular calcium chelating agent BAPTA‐AM. The results were consistent with W7 treatment, and BAPTA‐AM pretreatment restored the expression levels of LAP‐related proteins.

Finally, we attempted to investigate whether there is a correlation between the elevation of MAPK10 and the increase in calcium ion concentration. We pretreated THP1 cells with W7 and found that the elevation of MAPK10 induced by GT was restored by W7 (Figure S9). Thus, we speculate that the increase in MAPK10 may be caused by LAP inhibition and exists downstream of LAP.

## 4. Discussion

In this study, we demonstrated that GT inhibited LC3‐II conversion and the LAP process induced by *A. fumigatus* in THP1 cells; moreover, this inhibition was calcium‐dependent but not dependent on MAPK10. This is a new regulatory mechanism and function of GT, which might explain the possible inhibitory mechanism of GT on macrophage phagocytosis.

It was interesting to find out that exogenous GT significantly inhibited the conversion of LC3‐II in THP1 cells. Although GT has long been recognized for its immunosuppressive effect and negatively regulates a series of physiological signals in host cells, including the NF‐kB signaling pathway, its specific role and regulatory mechanism on infection are not clear. Recent studies have shown that GT promotes autophagy in macrophages during the infection of *Mycobacterium tuberculosis* [36]. This result seemed opposite to ours, which might be attributed to several reasons. First, the concentration of GT used in our study was 25 ng/mL, much lower than 10 μM GT (around equivalent to 80 ng/mL) used in that study. This is also confirmed by our results, as our use of 25 ng/mL GT did not affect cellular oxidative stress. In contrast, 50 ng/mL GT promotes upregulation of NOX2, p22phox, and p40phox expression and downregulation of GSH. As for why we chose a concentration of 25 ng/mL: First, our CCK‐8 assay results demonstrated that significant cell viability impairment only occurred at concentrations ≥100 ng/mL after 2 h of exposure, and the intentionally selected subtoxic concentrations (25/50 ng/mL) were chosen to ensure that the observed LAP‐related changes were not influenced by viability loss. Second, as evidenced in our previous research [15], a concentration of 20 ng/mL GT was sufficient to induce alterations in macrophage phagocytic activity, and the 25 ng/mL baseline concentration was selected to align with this established pathophysiological threshold while allowing for comprehensive dose‐response investigations. Furthermore, through an extensive literature review, we identified that the concentrations of bis(methylthio)gliotoxin (bmGT) and GT detected in the serum and bronchoalveolar lavage fluid (BALF) of patients with proven invasive aspergillosis (IA) typically do not exceed 50 ng/mL [37]. Therefore, our selection of concentrations below 50 ng/mL was designed to more accurately simulate physiological conditions in the human body, ensuring both biological relevance and scientific rigor in our investigation.

Additional evidence from western blotting and confocal microscopy further supported that the *gliP*Δ mutant, which was not able to generate GT, more significantly induced LAP, and this effect could be significantly inhibited by the addition of exogenous GT. These data support that GT does inhibit the autophagy response of macrophages in response to *A. fumigatus* conidia.

Because it was reported that there was a rather low amount of GT in the *A. fumigatus*‐contaminated environment and GT can be inhaled into the respiratory tract along with the conidia or probably produced by the colonized conidia or hyphae of *A. fumigatus* in the lung, its effect on innate immune cells might be of significance on host physiological reaction during the initial response against *A. fumigatus* or other pathogens. Furthermore, during the inhibition of LC3‐II conversion by GT, the expression of p62 also decreased synchronously. These two processes were basically consistent, indicating either the activation of autophagic clearance or a reduced formation of autophagosomes. Of course, further research is needed to determine whether it also has an inhibitory effect on autophagy caused by other spores or particles. As for VPS34, it makes up the PI3K complex with Beclin1, Vps15, UVRAG, and Rubicon. Oikonomou et al. [38] found that the lack of molecules required for LAP (Rubicon, Beclin‐1, NOX2, or Atg7) resulted in a decrease in LC3 recruitment to phagosomes, whereas they did not mention the individual role of VPS34, and VPS34 appears to play a role in targeting the complex to the LAPosome. Another study showed that inhibition of Vps34 did indeed interfere with the recruitment of LC3, but it did not clarify whether the changes in Vps34 were always consistent with autophagy levels [39]. Additionally, a study suggests that dendritic cell PIK3C3/Vps34 controls the pathogenicity of CNS autoimmunity independently of LAP [40]. In summary, there are various roles of Vps34 in autophagy regulation, and in our study, we did not further explore the specific mechanism of Vps34. In the future, we may investigate this further.

MAPK is activated and involved in regulating cellular immunity in many pathogen infection processes, including bacteria, fungi, and viruses [41], such as p38 MAPK, which plays an important role in neutrophil signaling in response to bacterial lipoproteins from *Staphylococcus aureus* [42]. In the present study, loss of GT in *A. fumigatus* conidia led to the decrease of MAPK10 expression in THP1 cells, and 25 ng/mL GT could stimulate MAPK10 expression to promote the generation of LC3‐II. This finding is consistent with our previous findings that *A. fumigatus* induced JNK expression and promoted autophagy in bronchial epithelial cells. However, this data hinted that GT might regulate the autophagy in host cells by two pathways. One, GT inhibits the LAP process; on the other hand, it might promote MAPK10 and LC3‐II conversion. These two pathways seemed to run in parallel or might keep a dynamic equilibrium that requires further experimental confirmation.

Another interesting finding in this study is that GT can significantly increase intracellular calcium ion concentration. However, there is currently no research on the effect of GT on calcium ions in host cells. This gap highlights an important area for future investigation, as understanding how GT influences calcium signaling could provide insights into its broader biological roles and potential therapeutic applications.

**Figure 6:**
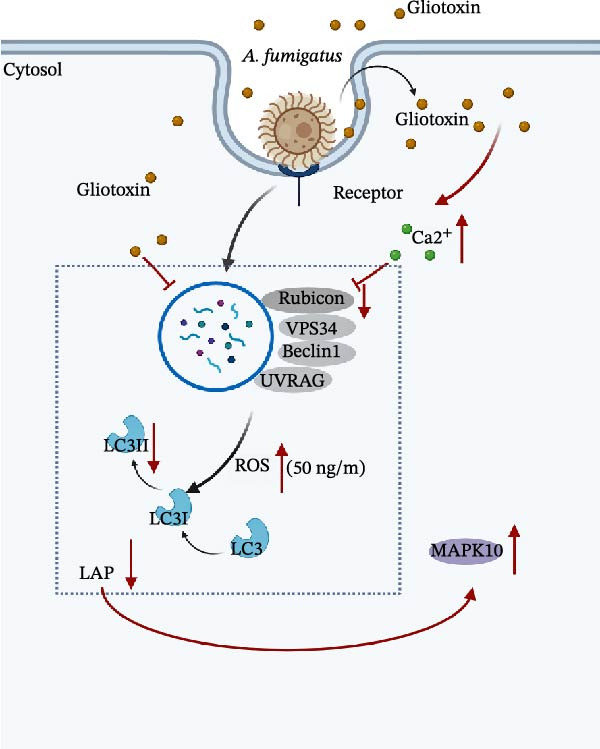
*A. fumigatus* induces LAP in macrophages, and in our research, (1) we demonstrated through WB and laser confocal microscopy that GT can inhibit the conversion of LC3‐II and the expression of Rubicon in a time‐dependent and dose‐dependent manner. (2) The results of flow cytometry and calcium ion fluorescence detection kits showed that GT promotes the release of calcium ions from macrophages, and calcium inhibition in turn restores LAP levels; we validated this by WB. (3) We found through the ROS detection kit that GT promotes ROS release at 50 ng/mL, but not at 25 ng/mL. (4) Finally, we demonstrated through WB and qPCR that MAPK10 exists downstream of LAP, and inhibition of calcium ions can reduce GT‐induced upregulation of MAPK10. (By Biorender).

Further, it was found here that the increase of calcium resulted in the depression of the LAP process. For this point, it is still skeptical on the relationship between intracellular calcium signaling and LAP, which is still questionable. Some reports indicated that the decrease of calcium leads to the prevention of LAP. For example, fungal melanin has been shown to sequestrate Ca^2+^–CaM signaling that depends on intracellular calcium ion sources from the endoplasmic reticulum, endoplasmic reticulum–phagosome association, and calcium release from the phagosome lumen, subsequently to prevent LAP [43]; In contrast, some study showed, in line with our findings that upregulation of mitochondrial calcium signaling inhibit autophagy as a survival strategy in *Mycobacterium tuberculosis* infection [35]. Although our data implicate calcium signaling as a key mediator of GT‐induced LAP suppression, alternative calcium‐dependent mechanisms warrant consideration. First, one potential mechanism involves the role of calcium/calmodulin‐dependent kinase (CaMK). The elevated calcium influx may stimulate the activation of CaMK and its downstream effector AMPK [44]; activated CaMK could interfere with phagosome acidification or lysosomal fusion, thereby inhibiting the completion of LAP [45, 46]. Additionally, the PKC family, a group of calcium‐dependent kinases, has been reported to regulate phagosome maturation through PKCα [47]. This process involves phosphorylation of NADPH oxidase components to promote ROS generation, a key trigger for LAP [48]. Studies have demonstrated that pathogen stimulation can induce calcium influx via the TLR4 pathway, activating the CaMKII‐Mst1/2‐Rac axis to enhance phagocytosis and mitochondrial‐phagosomal ROS production [49]. However, whether GT suppresses Rubicon expression by activating specific PKC isoforms through calcium signaling remains to be experimentally validated. In summary, the present study demonstrated for the first time that GT may lead to the impaired LAP of macrophages, which might be regulated by calcium signals but not by ROS and MAPKs. This provides new insight into the mechanism of pulmonary *A. fumigatus* infection (Figure 6).

However, this study has several limitations. Our findings are based on in vitro macrophage models, and validation in primary cells or in vivo systems will be necessary to further establish their physiological relevance. We employed three distinct macrophage lines to minimize cell‐line bias, yet future studies using primary macrophages and animal infection models would provide additional support. Moreover, this article focuses specifically on macrophages due to their central role in early antifungal defense. Whether GT exerts comparable effects on other immune cell types remains to be determined. Previous reports showing that GT impairs neutrophil phagocytosis and actin cytoskeleton organization suggest that additional innate immune populations may also be affected [17]. Further investigations are needed to define the broader immunomodulatory impact of GT during *A. fumigatus* infection.

## 5. Conclusion

Our data collectively show that GT inhibits LAP in macrophages through a calcium‐dependent mechanism, and the MAPK10 pathway exists downstream of LAP inhibition. However, our findings were based on the experiments in the cell lines in vitro.

In practice, studies on animals will be conducted to verify our results. This provides new insights for potential therapeutic strategies in treating IA.

**Nomenclature**
*A. fumigates*: *Aspergillus fumigatus*
GSH: Glutathione
GT: Gliotoxin
IPA: Invasive pulmonary aspergillosis
LAP: LC3‐associated phagocytosis
PLD: Phospholipase D
ROS: Reactive oxygen species.

## Author Contributions

Each author is expected to have made substantial contributions to the conception or design of the aricle. Conceptualization, methodology, visualization, data curation, and writing – original draft preparation: Yuqing Sun. Software and validation: Fangyan Chen. Formal analysis: Ling Zhang. Investigation: Caopei Zheng. Resources: Yu Wang. Writing– review and editing, supervision, project administration, funding acquisition: Yulin Zhang and Li Han.

## Funding

This work was supported by the National Natural Science Foundation of China (Grant 82172293 and 81571542) and Beijing Research Ward Excellence Program, BRWEP (BRWEP2024W042180100).

## Ethics Statement

The authors have nothing to report.

## Consent

The authors have nothing to report.

## Conflicts of Interest

The authors declare no conflicts of interest.

## Supporting Information

Additional supporting information can be found online in the Supporting Information section.

## Supporting information


**Supporting Information 1** Figure S1: Three types of *A. fumigatus* swollen conidia were cultured in a liquid medium for 6 h, and incubate swollen conidia with THP1 cells for 2 h, and take the cell culture supernatant for GT content detection. The results show that average GT content in the WT group was 31.36 ng/mL, the gliPR group was 32.05 ng/mL, and the *gliPΔ* group produced almost no GT.

**Supporting Information 2** Figure S2: (A, B) THP1 macrophages were treated with increasing concentrations of GT (0, 12.5, 25, 50, 100, and 200 ng/mL) for the indicated times (0–8 h). Cell viability was measured using a CCK‐8 assay, and absorbance was recorded at 450 nm with a microplate reader to evaluate dose‐ and time‐dependent cytotoxicity. (C, D) THP1 cells were treated with GT (25 ng/mL) for 2 h, and the F‐actin cytoskeleton was visualized using FITC‐labeled phalloidin (10 μM, 30 min, 37°C). Confocal microscopy revealed that GT treatment disrupted actin filament organization compared with untreated controls. All experiments were independently repeated at least three times.

**Supporting Information 3** Figure S3: (A, B) Confocal microscopy showed that after treating THP1 cells with 25 ng/mL GT for 2 h, the fluorescence of LC3 decreased. (C) Representative immunofluorescence images of LC3 in macrophages under different conditions: control, GT, infection with *A. fumigatus gliPΔ* swollen conidia (*gliPΔ*), *gliPΔ* conidia plus GT (*gliPΔ* + GT), and WT swollen conidia (WT). Nuclei are stained with DAPI (blue), conidia are shown in green,For groups without conidia (control and GT), fluorescent beads were added as size, and LC3 is shown in red. Compared with the control group, GT treatment reduced LC3 fluorescence, whereas WT conidia increased LC3 fluorescence. Infection with *gliPΔ* conidia further enhanced LC3 fluorescence compared with the WT group.

**Supporting Information 4** Figure S4: (A) mh‐s cells were treated with GT (0, 25, 50, 100 ng/mL) for 2 h, western blot analysis showed that GT inhibited conversion of LC3‐II in mh‐s cells in a dose‐dependent manner. (B) J774 cells were treated with GT (0, 25, 50, 100 ng/mL) for 2 h, western blot analysis showed that GT inhibited conversion of LC3‐II in J774 cells in a dose‐dependent manner. (C) mh‐s cells were treated with 25 ng/mL GT for 0, 30, 60, 120 min, western blot analysis showed that GT inhibited conversion of LC3‐II in mh‐s cells in a time‐dependent manner. (D) J774 cells were treated with 25 ng/mL GT for 0, 30, 60, 120 min, western blot analysis showedthat GT inhibited conversion of LC3‐II in mh‐s cells in a time‐dependent manner.

**Supporting Information 5** Figure S5: The volcano plot shows DEGs in THP1 macrophages infected with WT or *gliPΔ A. fumigatus* swollen conidia (MOI = 5) for 2 h. Red dots represent significantly upregulated genes, blue dots indicate downregulated genes, and gray dots correspond to non‐significant genes (threshold: |log_2_FC| ≥1, adjusted *p* < 0.05).

**Supporting Information 6** Figure S6: (A) THP1 macrophages were infected with WT or *gliPΔ* swollen conidia (MOI = 5) for 2 h. MAPK10 expression was assessed by qRT‐PCR. (B) THP1 macrophages were treated with GT (25 ng/mL) for 2 h. The relative mRNA expression of *MAPK10* was measured by qRT‐PCR. All experiments were independently performed at least three times.

**Supporting Information 7** Figure S7: THP1 macrophages were treated with GT (0, 25, 50, 100, and 200 ng/mL) for 2 h. Intracellular ROS production was quantified fluorometrically using a microplate reader. No significant differences in ROS production were observed across GT concentrations. All experiments were independently repeated at least three times.

**Supporting Information 8** Figure S8: THP1 macrophages were treated with GT (25 ng/mL, 2 h), calcium chelator BAPTA‐AM (10 μM, 1 h), or both. Western blot analysis and densitometric quantification showed that BAPTA‐AM partially reversed GT‐induced suppression of LC3‐II and Rubicon expression. All experiments were independently performed at least three times.

**Supporting Information 9** Figure S9: THP1 macrophages were treated with GT (25 ng/mL, 2 h), calcium inhibitor W7 (10 μM, 1 h), or both. Western blot analysis showed that GT upregulated MAPK10 expression, whereas W7 treatment partially attenuated this effect, suggesting that calcium signaling contributes to GT‐induced MAPK10 activation. All experiments were independently performed at least three times.

## Data Availability

The data that support the findings of this study are available from the corresponding author upon reasonable request.
