# Supplementary figures and images for: Aspergillus fumigatus Gliotoxin Inhibits LC3‐Associated Phagocytosis in Macrophages in a Calcium‐Dependent Manner
Source: J Immunol Res. 2026 Apr 29;2026:5542735. doi: 10.1155/jimr/5542735 (PMC13128990; doi:10.1155/jimr/5542735)

Supplemental Figure 1

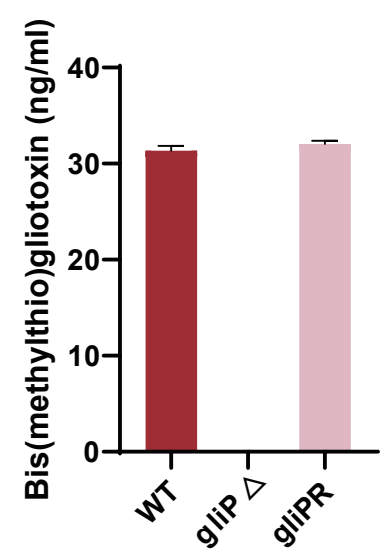

Supplement: Supplementary file 1 — Supporting Information 1 Figure S1: Three types of A. fumigatus swollen conidia were cultured in a liquid medium for 6 h, and incubate swollen conidia with THP1 cells for 2 h, and take the cell culture supernatant for GT content detection. The results show that average GT content in the WT group was 31.36 ng/mL, the gliPR group was 32.05 ng/mL, and the gliPΔ group produced almost no GT. [file JIMR-2026-5542735-s008.pdf]

Supplemental Figure 2

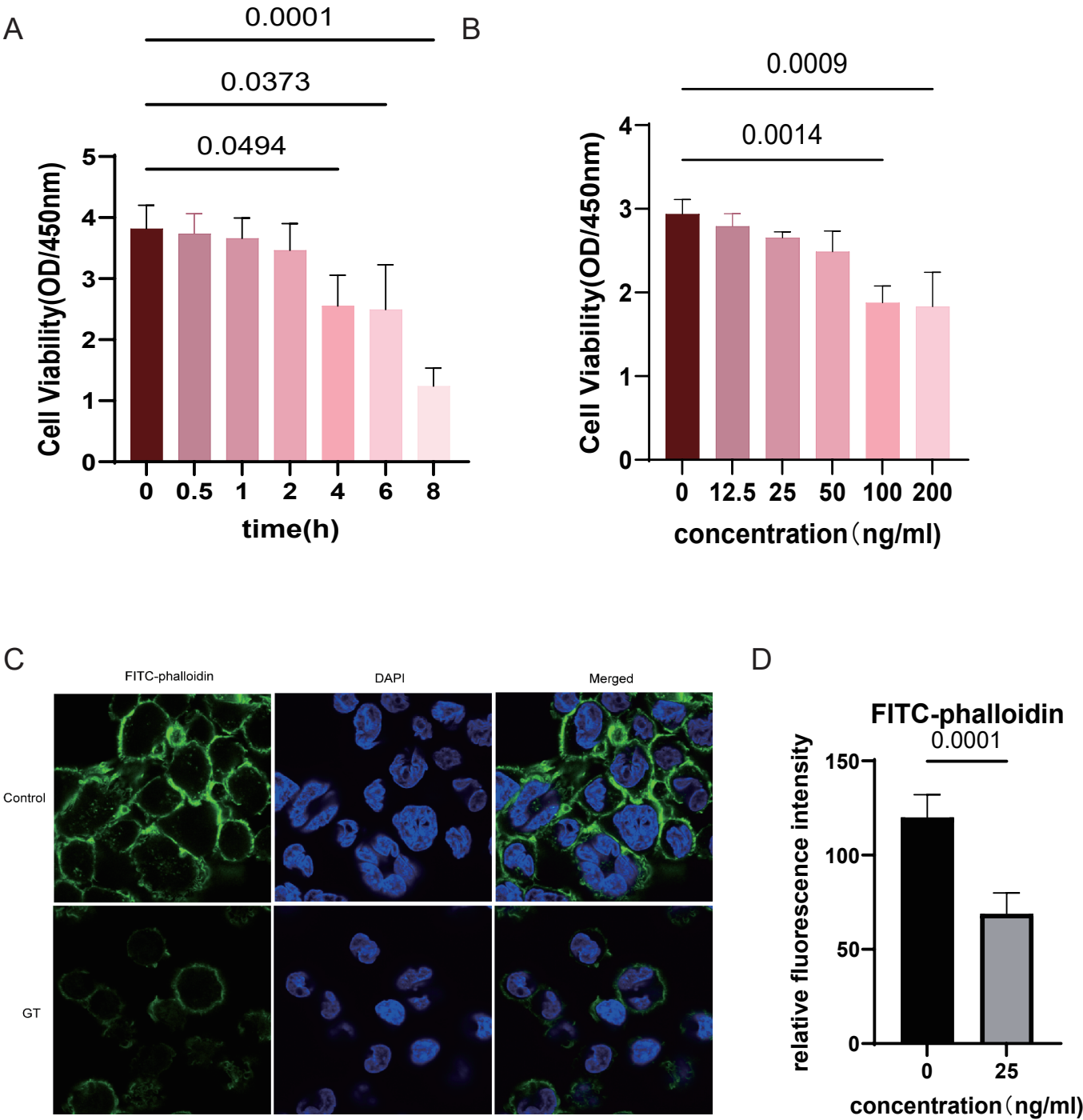

Supplement: Supplementary file 2 — Supporting Information 2 Figure S2: (A, B) THP1 macrophages were treated with increasing concentrations of GT (0, 12.5, 25, 50, 100, and 200 ng/mL) for the indicated times (0–8 h). Cell viability was measured using a CCK‐8 assay, and absorbance was recorded at 450 nm with a microplate reader to evaluate dose‐ and time‐dependent cytotoxicity. (C, D) THP1 cells were treated with GT (25 ng/mL) for 2 h, and the F‐actin cytoskeleton was visualized using FITC‐labeled phalloidin (10 μM, 30 min, 37°C). Confocal microscopy revealed that GT treatment disrupted actin filament organization compared with untreated controls. All experiments were independently repeated at least three times. [file JIMR-2026-5542735-s001.pdf]

Supplemental Figure 3

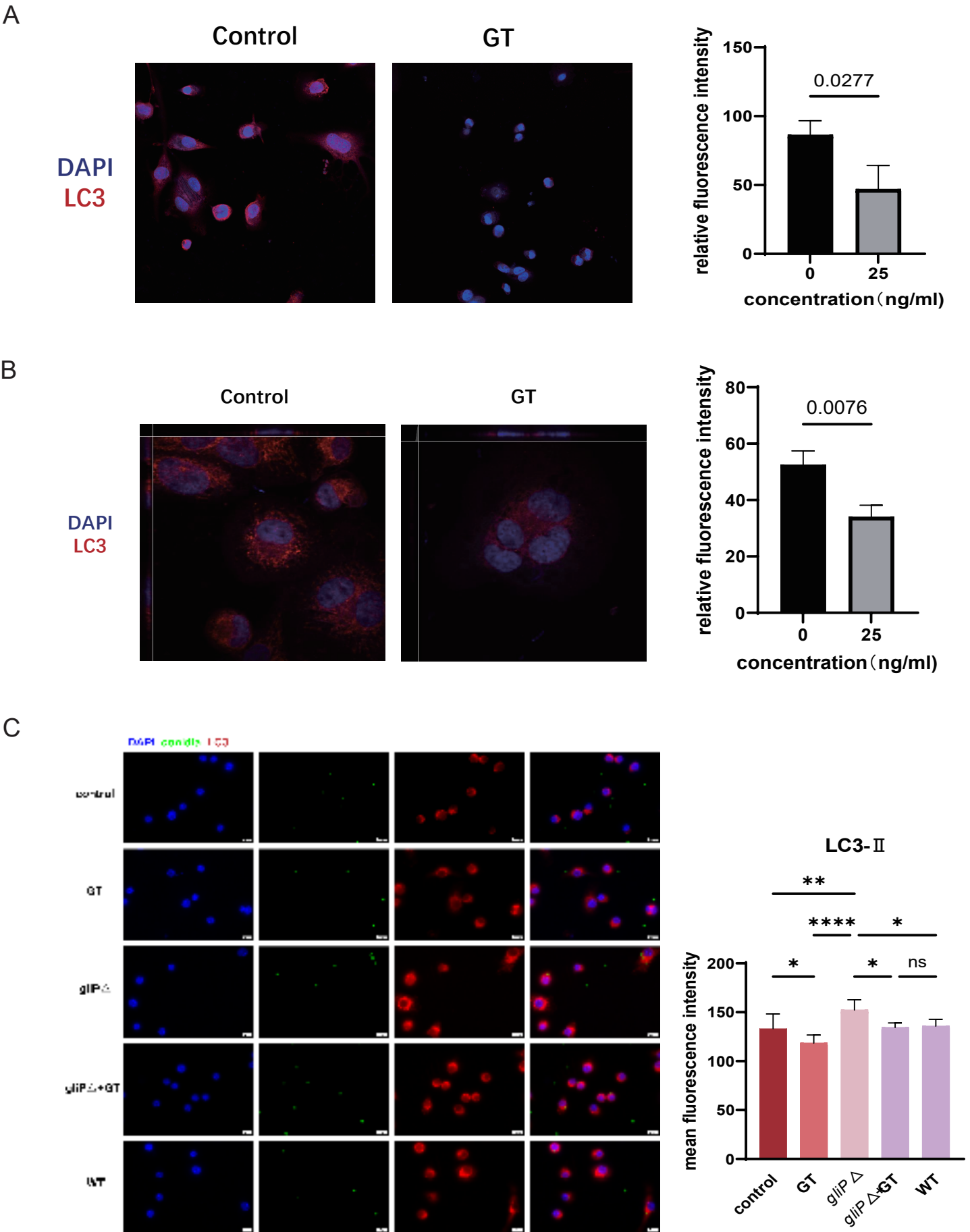

Supplement: Supplementary file 3 — Supporting Information 3 Figure S3: (A, B) Confocal microscopy showed that after treating THP1 cells with 25 ng/mL GT for 2 h, the fluorescence of LC3 decreased. (C) Representative immunofluorescence images of LC3 in macrophages under different conditions: control, GT, infection with A. fumigatus gliPΔ swollen conidia (gliPΔ), gliPΔ conidia plus GT (gliPΔ + GT), and WT swollen conidia (WT). Nuclei are stained with DAPI (blue), conidia are shown in green,For groups without conidia (control and GT), fluorescent beads were added as size, and LC3 is shown in red. Compared with the control group, GT treatment reduced LC3 fluorescence, whereas WT conidia increased LC3 fluorescence. Infection with gliPΔ conidia further enhanced LC3 fluorescence compared with the WT group. [file JIMR-2026-5542735-s002.pdf]

Supplemental Figure 4

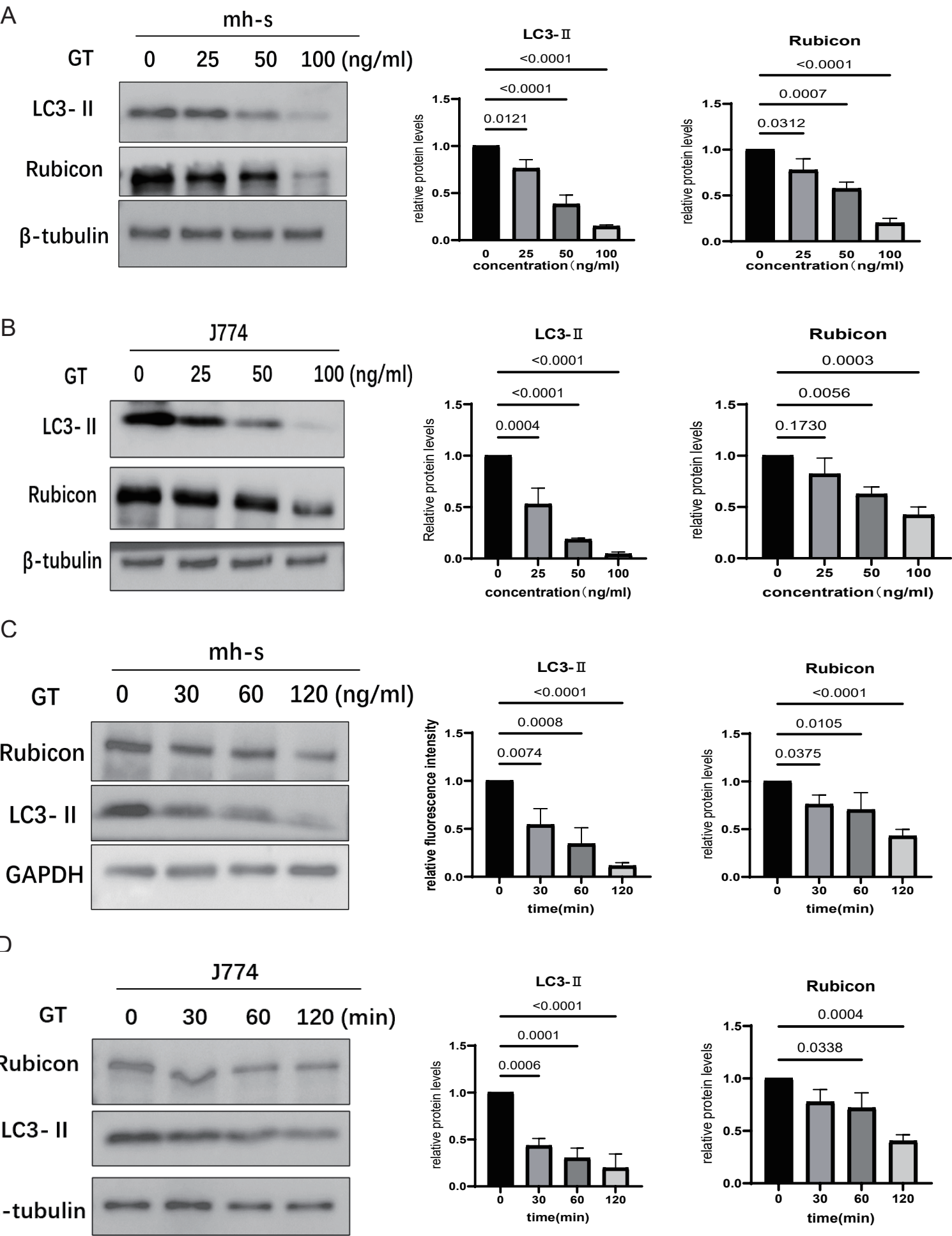

Supplement: Supplementary file 4 — Supporting Information 4 Figure S4: (A) mh‐s cells were treated with GT (0, 25, 50, 100 ng/mL) for 2 h, western blot analysis showed that GT inhibited conversion of LC3‐II in mh‐s cells in a dose‐dependent manner. (B) J774 cells were treated with GT (0, 25, 50, 100 ng/mL) for 2 h, western blot analysis showed that GT inhibited conversion of LC3‐II in J774 cells in a dose‐dependent manner. (C) mh‐s cells were treated with 25 ng/mL GT for 0, 30, 60, 120 min, western blot analysis showed that GT inhibited conversion of LC3‐II in mh‐s cells in a time‐dependent manner. (D) J774 cells were treated with 25 ng/mL GT for 0, 30, 60, 120 min, western blot analysis showedthat GT inhibited conversion of LC3‐II in mh‐s cells in a time‐dependent manner. [file JIMR-2026-5542735-s009.pdf]

Supplemental Figure 5

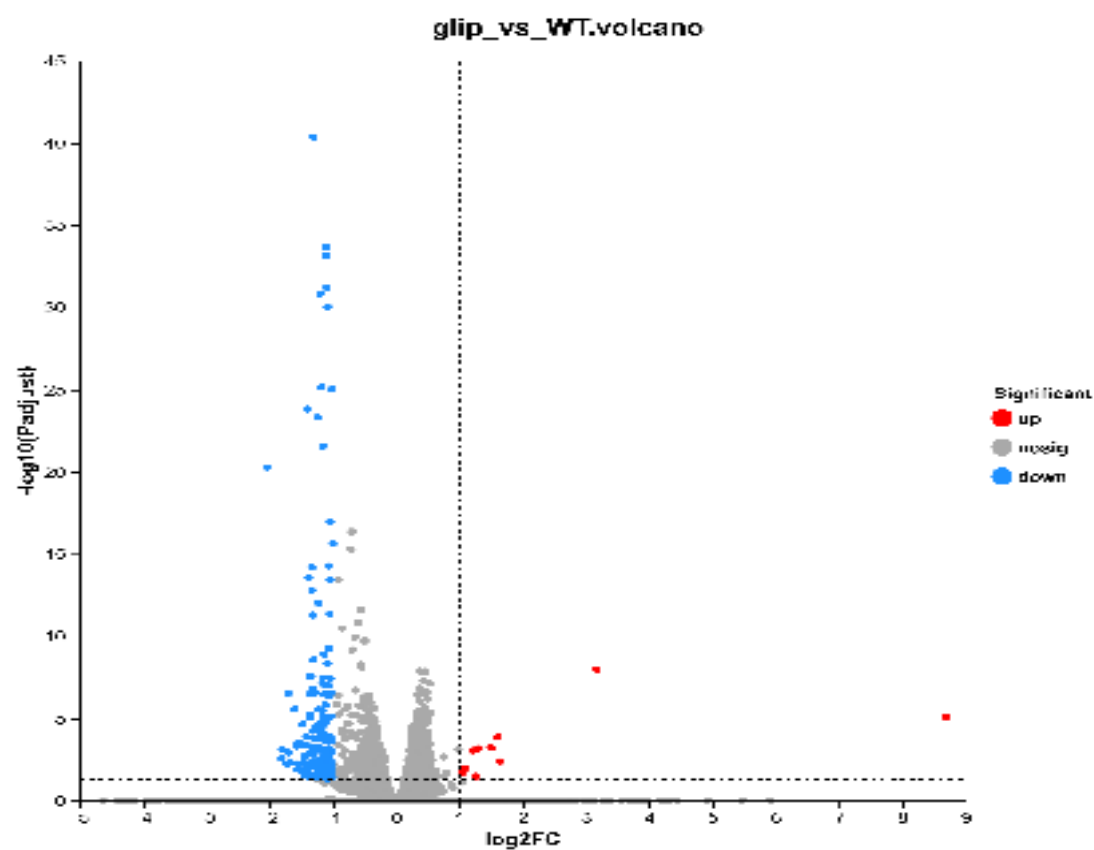

Supplement: Supplementary file 5 — Supporting Information 5 Figure S5: The volcano plot shows DEGs in THP1 macrophages infected with WT or gliPΔ A. fumigatus swollen conidia (MOI = 5) for 2 h. Red dots represent significantly upregulated genes, blue dots indicate downregulated genes, and gray dots correspond to non‐significant genes (threshold: |log2FC| ≥1, adjusted p < 0.05). [file JIMR-2026-5542735-s004.pdf]

Supplementary Figure 6

A

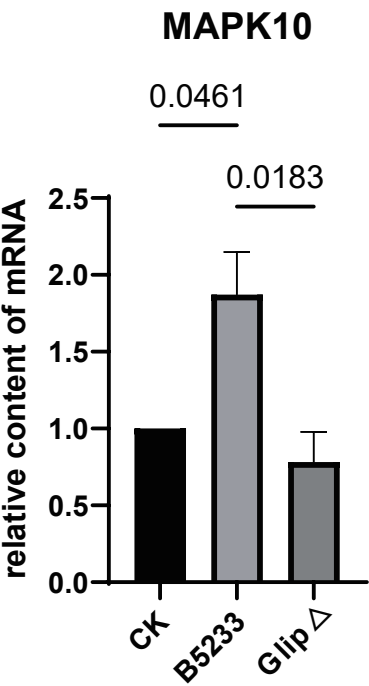

B

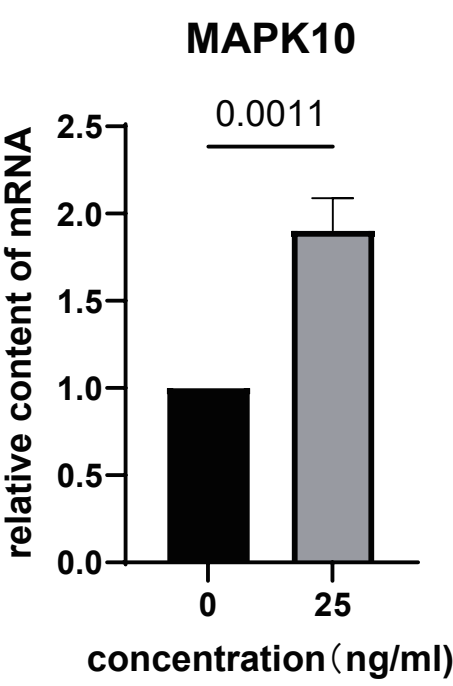

Supplement: Supplementary file 6 — Supporting Information 6 Figure S6: (A) THP1 macrophages were infected with WT or gliPΔ swollen conidia (MOI = 5) for 2 h. MAPK10 expression was assessed by qRT‐PCR. (B) THP1 macrophages were treated with GT (25 ng/mL) for 2 h. The relative mRNA expression of MAPK10 was measured by qRT‐PCR. All experiments were independently performed at least three times. [file JIMR-2026-5542735-s005.pdf]

Supplemental Figure 7

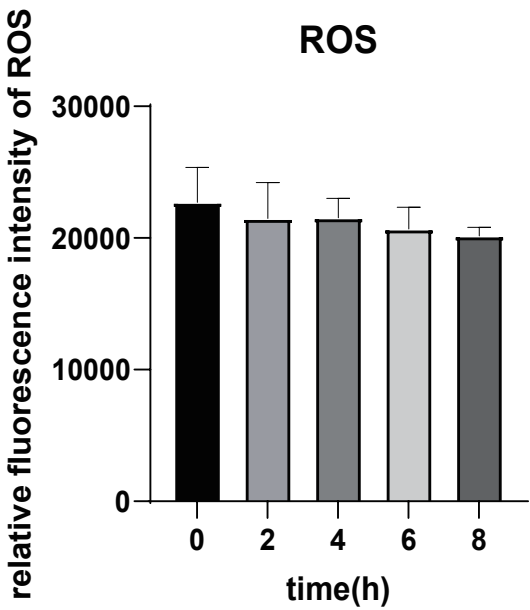

Supplement: Supplementary file 7 — Supporting Information 7 Figure S7: THP1 macrophages were treated with GT (0, 25, 50, 100, and 200 ng/mL) for 2 h. Intracellular ROS production was quantified fluorometrically using a microplate reader. No significant differences in ROS production were observed across GT concentrations. All experiments were independently repeated at least three times. [file JIMR-2026-5542735-s006.pdf]

supplemental Figure8

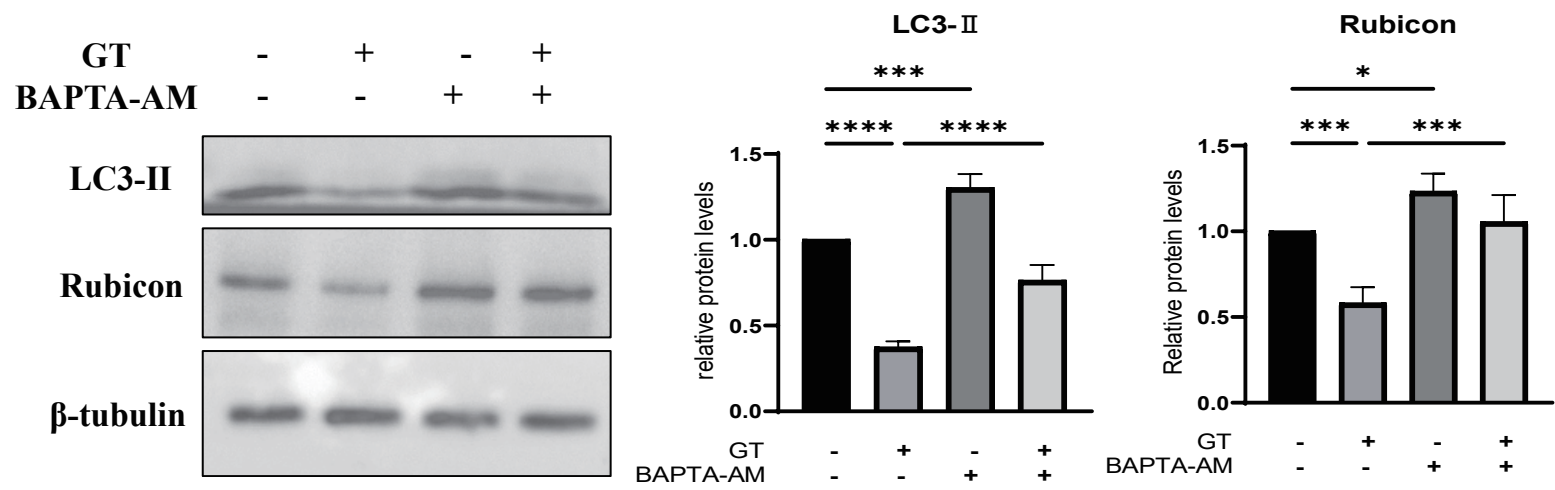

Supplement: Supplementary file 8 — Supporting Information 8 Figure S8: THP1 macrophages were treated with GT (25 ng/mL, 2 h), calcium chelator BAPTA‐AM (10 μM, 1 h), or both. Western blot analysis and densitometric quantification showed that BAPTA‐AM partially reversed GT‐induced suppression of LC3‐II and Rubicon expression. All experiments were independently performed at least three times. [file JIMR-2026-5542735-s007.pdf]

Supplemental Figure 9

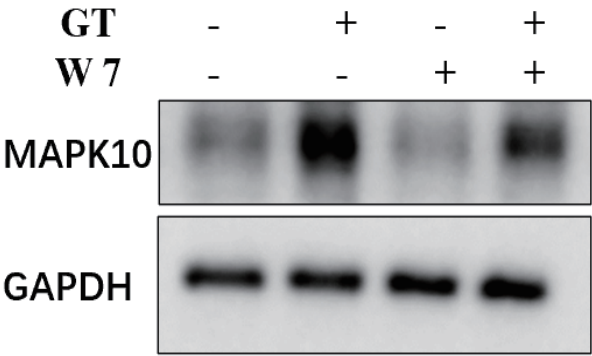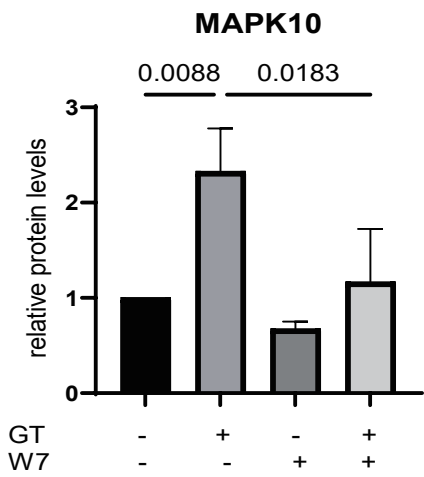

Supplement: Supplementary file 9 — Supporting Information 9 Figure S9: THP1 macrophages were treated with GT (25 ng/mL, 2 h), calcium inhibitor W7 (10 μM, 1 h), or both. Western blot analysis showed that GT upregulated MAPK10 expression, whereas W7 treatment partially attenuated this effect, suggesting that calcium signaling contributes to GT‐induced MAPK10 activation. All experiments were independently performed at least three times. [file JIMR-2026-5542735-s003.pdf]
